# Longitudinal associations between cardiorespiratory fitness and stress-related exhaustion, depression, anxiety and sleep disturbances

**DOI:** 10.1186/s12889-019-8081-6

**Published:** 2019-12-23

**Authors:** Agneta Lindegård, Gunilla Wastensson, Emina Hadzibajramovic, Anna Grimby-Ekman

**Affiliations:** 1 Institute of Stress Medicine, Region Västra Götaland, Carl Skottsbergs gata 22 B, 413 19 Gothenburg, Sweden; 2 Health Metrics, Community Medicine and Public Health, Sahlgrenska Academy, Gothenburg University, Gothenburg, Sweden; 3 Occupational and Environmental Medicine, Community Medicine and Public Health, Sahlgrenska Academy, Gothenburg University, Gothenburg, Sweden

**Keywords:** Cardiorespiratory fitness, Longitudinal associations, Stress-related exhaustion disorder, Sleep quality, Depression, Anxiety

## Abstract

**Background:**

In the last few years, so-called “common mental disorders”, including adjustment disorder and stress-related exhaustion, have outrivalled musculoskeletal disorders as being the leading cause of long-term sick leave in Sweden. Cardiorespiratory fitness level defined as “the maximal amount of physiological work that an individual can do as measured by oxygen consumption” has in many studies shown to reduce the risk of several life-style related diseases and moreover to improve mood, well-being and physical performance. The aim of the present study was to investigate, longitudinal associations between cardiorespiratory fitness and self-reported physical activity levels and the severity of symptoms connected to stress-related exhaustion, depression, anxiety, and sleep disturbances among women clinically diagnosed with stress-related exhaustion disorder (ED).

**Methods:**

The study was that of a longitudinal cohort study consisting of women (*n* = 88) diagnosed with stress-related ED in a specialist clinic in Gothenburg, Sweden. Cardiorespiratory fitness was measured with the Åstrand indirect test of maximal oxygen uptake (VO_2_max) and subjective measures of physical activity levels were rated on 4-graded physical activity scale. To measure and follow symptoms of ED over time the SMBQ-questionnaire (Shirom Melamed Burnout Questionnaire) was used. The Hospital Anxiety and Depression Scale (HADS) was used to measure depression and anxiety. A proxy variable for capturing overall disturbed sleep used to measure sleep. Longitudinal associations for continuous outcome variables and the dichotomous variable sleep were analysed using mixed- effects regression models with random intercepts. Regression coefficients along with the 95% confidence interval (CI) are presented as measures of association. Both exposures and the outcome were measured simultaneously over six waves (T1–T6).

**Results:**

The results showed statistically significant associations between level of fitness and reduced symptoms of stress-related exhaustion over time. Best improvements over time were seen in patients having a medium cardiorespiratory fitness level. No associations could be found between cardiorespiratory fitness level over time and anxiety, depression or sleep disturbances.

**Conclusion:**

Having medium cardiorespiratory fitness was positivity associated with a more sustained reduction in symptoms of ED overtime compared to those having low or high cardiorespiratory fitness levels. The clinical implication following this result is that an individual recommendation based on a medium level of physical activity in line with the recommendations from ACSM (American College of Sports Medicine) is preferable compared to recommendations including more vigorous physical activity in order to restore and sustainably reduce symptoms of exhaustion disorder over time.

## Background

In the last few years, so-called “common mental disorders”, including adjustment disorder and stress-related exhaustion, have outrivalled musculoskeletal disorders as being the leading cause of long-term sick leave in Sweden, according to official statistics from the Swedish Social Insurance Agency *(**https://www.forsakringskassan.se/wps/wcm/connect/8724a885-e6e8-4c07-aaf2-f087061b4103/socialforsakringen-i-siffror-2017.pdf?MOD=AJPERES&CVID=**)* [1]*.*

The diagnosis of stress-related exhaustion disorder, from here and onwards named ED, was established in Sweden in 2005, to better define and treat a subgroup of patients suffering from severe mental health problems caused by long-lasting identifiable stressors (International Statistical Classification of Diseases and Related Health Problems, 10th revision (IDC-10) code F43.8). The most salient characteristics of this patient group are extensive mental and physical exhaustion lasting for more than 6 months and caused by identifiable external stress exposures; sleep deprivation; decline in cognitive functions; and, in most cases, psychiatric and somatic co-morbidity [2, 3]*.* The most frequent psychiatric disorders accompanying ED are depression and different kinds of anxiety diagnoses including generalized anxiety disorder (GAD) [2]. From earlier studies in patients with these symptoms it is also known that sleep disturbances are highly prevalent among in these patients. Moreover, sleep problems are often the main reason for seeking medical care. The association between lack of sleep and/or poor sleep quality, high perception of stress, and common mental disorders including ED has been established in several previous studies [4–10].

According to the existing scientific literature, many of the symptoms experienced in connections with stress-related exhaustion and co-morbid depression and anxiety are associated with physical inactivity [11, 12]. H*ence, one of the leading treatment strategies in clinical practice has been to incorporate increased levels of physical activity/exercise as part of the basic treatment for this patient group. Regarding symptoms of depression and possible associations with different levels of physical activity, there is a growing body of evidence for the importance of increased and sustained physical activity levels as part of the basic treatment for patients suffering from depression* [11, 13, 14]*. However,* studies exploring the existence of similar associations and effects of physical activity in patients diagnosed with ED are still scarce. In a previous study by our research group, we found that the current recommendations of 150 min of physical activity per week, also stated by the American College of Sports Medicine (ACSM), were sufficient to significantly decrease symptoms of stress-related exhaustion and depression among patients with ED [15]. D*espite the huge interest from both researchers and practitioners to explore and further strengthen the evidence for the positive effects of physical activity interventions among patients with ED, there is still a lack of good-quality longitudinal studies confirming this positive association. The few studies performed in these patients (including our own study, cited above) have until now mostly relied on self-reported data, including our own previous study; therefore, more studies using objective physical activity measures are highly warranted.*

*T*he aim of the present study was thus to investigate longitudinal associations between objectively measured cardiorespiratory fitness levels and the severity of symptoms of exhaustion disorder (ED), depression, anxiety, and sleep disturbances in women clinically diagnosed with ED. Furthermore, to investigate the same longitudinal associations between self-reported physical activity levels regarding the same outcome variables in the same study group.

## Methods

### Study design and procedures

The study had a longitudinal design and was conducted in a closed cohort of women diagnosed with ED by two experienced physicians. The reason for not having any men in the cohort was that by that time (2006), when we started to include patients most of our patients were women (75%), as were the situation for most clinics treating patients with stress-related ED. Thus, to include both men and women who fulfilled the rather rigorous criteria would have taken by far too long time. Data was collected on several occasions, at baseline and follow-up after 3, 6, 9, 12 and 18 months, between the years 2006–2010 at the Institute of Stress Medicine in Gothenburg, Sweden. Both at baseline and during follow-ups, sleep disturbances, symptoms of ED, depression and anxiety, as well as physical activity level was measured. The level of physical activity was measured using both an objective method and a self-reported method, on each measuring occasion. During the patients’ first appointment with the physician the medical diagnosis “exhaustion disorder (ED)” (IDC-10 code F43.8) was set by means of an especially elaborated questionnaire in combination with a clinical interview and examination, performed by either of the two specialist doctors engaged in the study. Besides being diagnosed with stress-related exhaustion, the inclusion criteria for entering the study were as follows: age between 25 and 64 years and having had less than 6 months’ sick leave due to current symptoms of stress-related exhaustion/burnout. The exclusion criteria were: ongoing infection, anemia, diabetes, alcohol abuse, vitamin B12 deficiency, thyroid disorders and/or any other disorder (e.g. cardiovascular disorders) or side effects of medication that could affect the interpretation of the results.

### Participants

Since early 2004, general practitioners, specialist clinics and company doctors in the region of Västra Götaland have been able to refer patients with stress-related exhaustion disorders to a specialist clinic, the Institute of Stress Medicine (ISM), where they have received specialist care from a team comprising medical doctors, psychologists, nurses and physiotherapists. The aim of this clinic, besides treating patients, is also to perform research on the development and progression, prevention, and treatment of the disorder. Consequently, most of the patients referred to the clinic have participated in different studies conducted at the clinic. Regarding all studies, including the present one, only patients meeting the criteria for ED have been included; hence, all 88 patients in our study was originally included in a randomized controlled trial (RCT) with the aim of exploring the effects of either manualized cognitive behavioural therapy (MCBT) (nine sessions) or coached physical activity (26 weeks) on recovery from ED/burnout. Hence, the sampling frame for this here study was the same as for the original RCT study. The data collection started in 2006 and ended in 2010. According to the study protocol for the randomised controlled trial all 88 patients initially received a basic treatment. The main components of this basic treatment consisted of one interactive group session (2-h) explaining and discussing the known causes and consequences of long-lasting stress exposure, on both mental as well as somatic health in general. In addition, all patients were invited to take part in a group-sessions in “stress- management” once a week for 7 weeks. All patients were also offered consultations with the physician at intervals of 4–6 weeks for as long as 12 months after the first consultation. Importantly, during the visits, the physicians placed great emphasis on discussing lifestyle-related topics (including the relevance of regular physical activity) with the patients. Due to methodological problems, the originally RCT-study was never carried out in full. However, data relevant for this here study was continuously collected in our patient register.

As mentioned before, our study group consisted of 88 women with a mean age of 44 (range 26–64) years. Background characteristics for the study group, such as level of education (where high education corresponds to university studies and low education therefore corresponds to no university education), perceived sleep disturbances (measured using the Karolinska Sleep Questionnaire (KSQ), smoking habits, body mass index (BMI), alcohol consumption (measured with the Alcohol Use Disorders Identification Test (AUDIT), self-rated symptoms of depression and/or anxiety (measured by the Hospital Anxiety and Depression Scale (HADS) and self-rated ED (measured with the Shirom-Melamed Burnout Questionnaire (SMBQ) are presented in Table 1.

**Table 1:** Baseline data for all participants (*n* = 88)

Characteristics	N		Median (IQR)
Mean age, years (range)	88	44 (26–64)	43 (12)
Education	87		
high, % (n)		67% (58)	
low, % (n)		33% [29]	
Mean body mass index (BMI) (range)	88	24.2 (18.0–33.0)	24.1 (3.8)
Current smokers/nicotine users, % (n)	87	14% [12]	
Mean AUDIT score (range)	88	2.7 (0–12)	2.0 (2.0)
Symptom duration, % (n)	86		
≥ 1 year		51% [44]	
< 1 year		49% [42]	
Use of antidepressants, % (n)	88	28% [25]	
KSQ insomnia index (range)	79	3.0 (1.0–5.8)	3.0 (1.5)
KSQ sleepiness index (range)	75	4.1 (1.0–5.8)	4.4 (1.0)
KSQ premature awakening index (range)	79	2.9 (1.0–6.0)	3.0 (1.7)
KSQ single item, poor sleep quality, % (n)	78	68% [53]	
HADS depression sum score (range)	88	9.5 (1.0–19)	9.0 (6.0)
HADS anxiety sum score (range)	88	12 (0–20)	12 (7.0)
SMBQ sum score (range)	83	5.6 (3.0–6.9)	5.6 (1.2)
SMBQ > 4.4, % (n)	83	95% (79)	

AUDIT = Alcohol Use Disorders Identification Test; HADS = Hospital Anxiety and Depression Scale; KSQ = Karolinska Sleep Questionnaire; SMBQ = Shirom-Melamed Burnout Questionnaire

### Measures

#### Cardiorespiratory fitness

Cardiorespiratory fitness was measured with the Åstrand indirect test of maximal oxygen uptake (VO_2_max) [16, 17]. This submaximal test was performed in the morning (starting at 7:00 am, 7:30 am, or 8:00 am) on a bicycle ergometer (Monark Ergomedic 828E, Monark Exercise AB, Vansbro, Sweden). A submaximal aerobic capacity test was used because of its ability to measure fitness among patients with poor health and low fitness levels. The pedalling frequency was 50 rounds per minute (rpm), and the workload was adjusted to keep the heart rate at 130–160 beats per minute (bpm) in participants < 40 years old and 120–150 bpm in participants ≥40 years old. Perceived exertion during the test was rated on a Borg scale [18], and the patients were advised to try to keep their exercise intensity level at 13 or 14 (slightly strenuous). A steady state was reached when the heart rate remained stable after 5 or 6 min, or whenever a stabilization occurred afterwards. A nomogram based on sex, workload, and mean steady-state heart rate was used to estimate the peak oxygen uptake (L/min) [19]. Additionally, a correction factor for age was used, and the oxygen uptake was corrected for body weight and then expressed as peak VO_2_max (mL/kg/min), which is considered a valid estimate of cardiorespiratory fitness [20]. At baseline all included patients were categorized into three groups based on their test values: a low cardio respiratory fitness group, a medium cardio respiratory fitness group and a high cardiorespiratory fitness group, according to Åstrand et al. [19]. This cardiorespiratory fitness test was performed at baseline and at each follow-up occasion (3,5,12 and 18 months).

Data collection regarding self-reported physical activity level was conducted using an adapted version of the commonly used 4-graded physical activity scale developed by Saltin and Grimby already in 1968 [21]. The validity of this scale was recently scrutinized with respect to health research, and the predictive validity was found to be good for various risk factors for different health conditions, as well as for morbidity and mortality [22]. The scale consists of one question: “How much do you move and exert yourself physically during your leisure time? (If your activity varies greatly between, for example, summer and winter, try to estimate an average. The question refers to the past year.)” The four response alternatives were 1 = mostly sedentary (almost completely inactive, reading, watching television, watching movies, using computers or doing other sedentary activities during leisure time); 2 = some light physical activity (physically active for at least 4 h/week, such as riding a bicycle or walking to work, walking with the family, gardening, fishing, table tennis, bowling, etc); 3 = regular physical activity and training (doing heavy gardening, running, swimming, playing tennis or badminton, doing calisthenics and similar activities, for at least 2–3 h/week); 4 = regular hard physical training for competitive sports (running, orienteering, skiing, swimming, playing football, handball, eg several times per week for at least 5 h/week).

In line with the procedure for the cardiorespiratory fitness test the patients were categorized into three groups where response alternative 1 (mostly sedentary) comprised one group, alternative 2 (light physical activity) formed another group and response alternatives 3 and 4 (more intensive and high intensity activity) constituted the third group. The classification was done according to a previous epidemiological study on group level using the same instrument [23]*.*

Self-reported physical activity data were collected at baseline and at each follow up occasion (3, 6 12 and 18 month).

#### Symptoms of stress-related exhaustion (ED)

To measure and follow symptoms of ED over time the SMBQ-questionnaire (Shirom Melamed Burnout Questionnaire) was used [24]. This instrument has previously been used to follow the course of symptoms of ED in a similar patient group (Glise et al. 2012). The reason for selecting this instrument relates to fact clinical burnout and ED scare many core elements with respect to symptomatology, and the two diagnoses have in previous literature been regarded as highly interrelated ( [5, 25].

The SMBQ originally contained 22 items with four subscales: physical fatigue (eight items), cognitive weariness (six items), tension (four items), and listlessness (four items). In the present study, a revised 18-item version (the “tension” dimension excluded) was used and proved to have good construct validity. All items are expressed as statements and are rated using a 7-point response scale (ranging from *almost never* to *almost always*). Instead of the mean score for the 18 items, a recommended transformed score is calculated [26]. This score ranges from 18 to 126, with higher values indicating higher degree of burnout, and with a 79-point raw score, corresponding to the recommended 4.4 cut-off for clinical burnout/ED [26].

Moreover, the SMBQ instrument has been demonstrated to correlate highly with other reliable burnout instruments, such as the Maslach Burnout Inventory and Pines Burnout Measure [27]*.*

#### Anxiety and depression

The Swedish version of the Hospital Anxiety and Depression Scale (HADS) was originally used to assess the prevalence and severity of different states of depression and anxiety in the setting of a hospital medical outpatient clinic [28, 29]. The scale is divided into two parts, one part that measures level of anxiety and the other measuring level of depression. The questions were originally designed for non-psychiatric patients to detect states of symptoms of depression and anxiety [29]. The HADS consists of 14 statements concerning feelings during the past week, seven for each of the two subscales. Four response alternatives (scored 0–3) indicating degree or frequency of symptoms/feelings are available for each statement. In this study, analysis of Rasch-transformed scores ranging from 0 to 21 was used, with higher scores indicating higher levels of depression, respectively anxiety. The HADS has previously been found to have satisfactory factor structure and internal consistency, as well as acceptable discriminant and concurrent validity, in a variety of clinical settings [30].

#### Sleep disturbances

To measure sleep disturbances we used a previously identified proxy variable for capturing overall disturbed sleep “How do you perceive your sleep as a whole?” with five response alternatives, where 1 = *very poor*, 2 = *fairly poor*, 3 = *neither good nor poor*,4 = *fairly good*, and 5 = *very good*, as recommended by Söderström and co-workers [31]. The answers were then dichotomized into one “poor sleep” category including the responses *very poor* and *fairly poor* sleep and one “good sleep category” including the responses *neither good nor poor* sleep, *fairly good* sleep, and *very good* sleep.

#### Data treatment and statistical analysis

Median values and interquartile ranges were used to characterize the study group at baseline. When stratifying groups by both self-reported and objectively measured level of fitness, the Kruskal-Wallis and Fisher’s exact tests were used to compare outcome measures and possible confounders at baseline. Additional pairwise comparisons were performed using nonparametric tests (Wilcoxon rank sum test and Fisher’s exact test). Longitudinal associations for continuous outcome variables were analysed using mixed- effects regression models with random intercepts only model. Longitudinal associations for the dichotomous outcome variable sleep were likewise analysed using mixed-effects logistic regression models with random intercepts only model. Regression coefficients along with the 95% confidence interval (CI) are presented as measures of association. Both exposures and the outcome were measured simultaneously over six waves (T1–T6). All analyses were accomplished by means of the software program SAS version 9.4 (SAS Institute, Cary, NC, USA) and SPSS version 22.0 (IBM, Armonk, NY, USA) [32]. In the regression models possible confounders were included if *p* < 0.25. The possible confounders tested were education level, use of anti-depressive medication at baseline, smoking habits, BMI, symptom duration, use of alcohol, self-rated physical activity level, and age.

##### Ethical review board

This study was approved by the Regional Ethical Review Board, Gothenburg, Sweden, in 2006-08-07 (number 398–06). Written consent from all participating patients was included in the ethical approval.

## Results

### Baseline data cardiorespiratory fitness

The mean age for the entire study group (*n* = 88) was 44 (range 26–64) years. Nearly all patients (95%) scored above the clinical level for severe stress-related exhaustion/burnout using the SMBQ instrument. The average time for symptom duration was equally distributed between less than 1 year (49%), and 1 year and longer (51%) (Table 1). In Table 1 other relevant descriptive characteristics for the whole study group are shown such as body mass index, self-rated sleep quality, use of anti-depressive medication etc. When we stratified the study group into the three categories “low fitness”, “medium fitness” and “high fitness” based on cardiorespiratory fitness test at baseline, data revealed significant inter-group differences regarding age, education level and BMI (Table 2). Further analyses showed that patients with low fitness level at baseline were significantly younger, had a lower education level and higher BMI compared with their counterparts with high fitness level at baseline (data not shown). Regarding the outcome variables burnout, anxiety, depression and sleep disturbances, no significant differences regarding severity of symptoms were found between the three fitness levels at baseline.

**Table 2:** Baseline data by level of cardiorespiratory fitness (*n* = 79)

	Low fitness (*n* = 20)			Medium fitness (*n* = 26)			High fitness (*n* = 33)			
*Characteristics*	N	Median	IQR	N	Median	IQR	N	Median	IQR	*p*-value
Age, yrs.	20	39	7.0	26	43	8.0	33	48	10	0.001^a^
Education	20			26			32			0.03^b^
high, % (n)		45% [9]			65% [17]			81% [27]		
slow, % (n)		55% [11]			35% [9]			19% [5]		
Body mass index (BMI)	20	25.5	5.1	26	24.1	3.0	33	23.1	3.8	0.01^a^
Current smokers/nicotine users, % (n)	20	10% [2]		26	12% [3]		32	19% [6]		0.65^b^
AUDIT score	20	3.0	2.5	26	2.0	3.0	33	2.0	2.0	0.60^a^
Symptom duration, % (n)	19			26			32			0.27^b^
≥ 1 year		37% [7]			46% [12]			59% [19]		
< 1 year		63% [12]			54% [14]			41% [13]		
Use of antidepressants, % (n)	20	35% [7]		26	27% [7]		33	30% [10]		0.91^b^
KSQ insomnia index	17	2.8	0.8	24	3.0	1.3	31	2.8	2.3	0.51^a^
KSQ sleepiness index	16	4.2	1.4	23	4.0	1.2	29	4.4	0.6	0.24^a^
KSQ premature awakening index	18	2.8	2.0	24	2.5	2.0	30	3.2	1.3	0.85^a^
KSQ single item, “poor sleep” quality, % (n)	18	83% [15]		23	52% [12]		30	67% [20]		0.12^b^
HADS depression sum score	20	10	7.0	26	9.5	9.0	33	9.0	5.0	0.57^a^
HADS anxiety sum score	20	13	6.0	26	13	5.0	33	11	7.0	0.69^a^
SMBQ sum score	18	5.6	1.0	25	6.0	1.5	32	5.5	1.0	0.51^a^
SMBQ > 4.4, % (n)	18	100% [18]		25	88% [22]		32	97% [31]		0.21^b^

^a^Kruskal-Wallis test; ^b^Fisher’s exact test*.* AUDIT = Alcohol Use Disorders Identification Test; HADS = Hospital Anxiety and Depression Scale; KSQ = Karolinska Sleep Questionnaire; SMBQ = Shirom-Melamed Burnout Questionnaire

### Baseline data self-reported physical activity

In concordance with the second aim of this study we also stratified the study group, based on self-reported physical activity data, into three categories, patients with a “low physical activity level”, a “medium physical activity” level and a “high physical activity” level (Table 3). The three categories showed significant differences regarding age and symptom duration. Further analyses revealed that patients characterized by low physical activity level had significantly longer symptom duration compared with those having a medium physical activity level, and furthermore there was a significant difference in age between patients characterized with a high physical activity level and those characterized as having a medium physical activity level (data not shown). There were also differences at baseline regarding the two outcome variables sleepiness and premature awakening (Table 3), and further analyses revealed that the “high physical activity group” reported less sleep disturbances compared with the other two groups (data not shown). However, no significant difference was found between groups regarding sleep disturbances in general (the single item question previously described in the method section). Finally, a significant difference was seen between the different physical activity levels with respect to the perceived burden of burnout symptoms. Those with a low physical activity level had more symptoms of stress-related exhaustion/burnout compared with the other levels at baseline; however, initially, as mentioned before, almost all participants scored above the cut-off for severe clinical burnout.

**Table 3:** Baseline data by self-reported physical activity level (*n* = 83): the “low physical activity”, “medium physical activity” and “high physical activity” level

	Low fitness			Medium fitness			High fitness			
*Characteristics*	N	Median	IQR	N	Median	IQR	N	Median	IQR	*p*-value
Age, yrs.	13	43	5	49	41	7.0	21	48	13	0.04^a^
Education	13			48			21			0.68^b^
high, % (n)		77% [10]			63% [30]			67% [14]		
low, % (n)		23% [3]			37% [18]			33% [7]		
Body mass index (BMI)	13	21.9	5.1	49	24.5	4.1	21	23.7	2.7	0.07^a^
Current smokers/nicotine users, % (n)	13	8% [1]		48	15% [7]		21	19% [4]		0.75^b^
AUDIT score	13	2.0	4.0	49	2.0	2.0	21	3.0	1.0	0.41^a^
Symptom duration	13			47			21			0.02^b^
≥ 1 year		85% [11]			40% [19]			57% [12]		
< 1 year		15% [2]			60% [28]			43% [9]		
Use of antidepressants, % (n)	13	31% [4]		49	33% [16]		21	19% [4]		0.59^b^
KSQ insomnia index	12	2.9	1.9	48	3.0	1.3	19	2.8	1.5	0.54^a^
KSQ sleepiness index	11	3.8	1.6	47	4.2	1.0	17	4.8	1.2	0.04^a^
KSQ premature awakening index	13	1.7	2.3	48	2.7	1.7	18	3.7	1.7	0.01^a^
KSQ single item, poor sleep quality, % (n)	13	69% [9]		47	68% [32]		18	67% [12]		0.99^a^
HADS depression sum score	13	13	6.0	49	9.0	7.0	21	9.0	5.0	0.10^a^
HADS anxiety sum score	13	14	8.0	49	12	7.0	21	11	5.0	0.50^a^
SMBQ sum score	12	6.5	0.9	45	5.6	1.1	21	5.2	1.0	0.002^a^
SMBQ > 4.4, % (n)	12	100% [12]		45	96% [43]		21	90% [19]		0.62^b^

^a^Kruskal-Wallis test; ^b^Fisher’s exact test. AUDIT = Alcohol Use Disorders Identification Test; HADS = Hospital Anxiety and Depression Scale; KSQ = Karolinska Sleep Questionnaire; SD = standard deviation; SMBQ = Shirom-Melamed Burnout Questionnaire

### Follow-up

#### Cardiorespiratory fitness and symptoms of ED

**Fig. 1:**
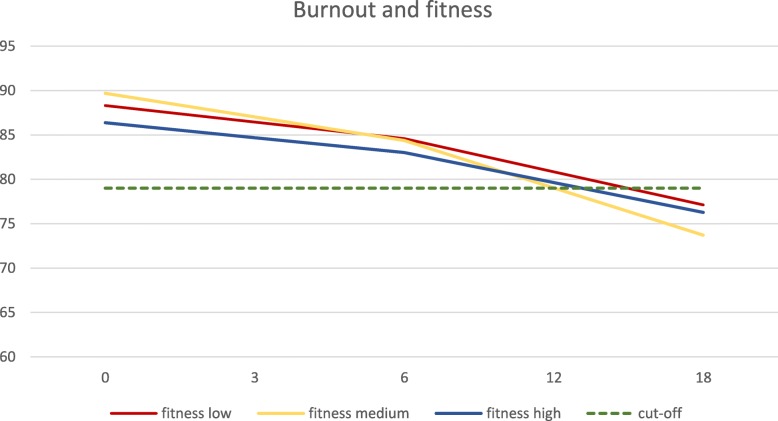
Longtudinal associations between symptoms of burnout, measured using the Shirom-Melamed Burnout Questionnaire (SMBQ), and different cardiorespiratory fitness levels. The clinical cut-off, at SMBQ = 79, is shown

The results showed statistically significant associations between level of cardiorespiratory fitness and symptoms of ED over time when we adjusted for the two variables symptom duration and use of antidepressants (Table 4). Moreover, the effect of fitness level is different over time (the interaction between time and fitness was significant, type III *p* = 0.047). At baseline, the level of burnout in patients with low fitness level was 88.3, in patients with medium fitness 89.7 and in patients with high fitness 83.4. At the 18 months follow-up the corresponding levels were 77.1, 73.7 and 76.3 respectively, see Fig. 1. All three levels scored below the clinical cut-off level 4.4 (raw score 79 on the revised 18 item scale) at the 18-month follow-up. Thus, the best improvements in burn-out score in the long-term perspective was seen in patients with a medium cardiorespiratory fitness level (Fig. 1).

**Table 4:** Mixed-effects regression analysis of the association between fitness level (high, medium, low) and time with respect to clinical burnout, depression and anxiety, adjusted for symptom duration and use of anti-depressive medication

	Burnout			Depression			Anxiety		
	Coeff	95% CI	Type III *p*-value	Coeff	95% CI	Type III *p*-value	Coeff	95% CI	Type III *p*-value
*Intercept*	94.4	90.46; 98.35	< 0.0001	10.7	9.54; 11.84	< 0.0001	10.7	9.85; 11.65	< 0.0001
*Time*	−0.6	−0.83; −0.32	< 0.0001	−0.2	−0.26; −017	< 0.0001	−0.2	− 0.25; − 0.16	< 0.0001
*Fitness*			0.030			0.075			0.252
High	−2.0	−5.58; 1.48		−1.2	−2.26; −0.17		− 0.7	−1.71; 0.22	
Medium	1.8	−1.62; 5.28		−0.7	−1.67; 0.27		−0.7	− 1.58; 0.22	
Low	0			0			0		
*Symptom duration*			0.002			0.030			
< 1 year	−4.9	−7.94; −1.90		−1.3	−2.51; −0.14				
≥ 1 year	0			0					
*Antidepressant use*			0.006						
No	−4.8	−8.27; −1.42							
Yes	0								
*Interaction*			0.047						
Time*high	0.01	−0.30; 0.32							
Time*medium	−0.3	−0.67; 0.01							
Time*low	0								

CI = confidence interval; coeff = coefficient

#### Cardiorespiratory fitness depression and anxiety

In the longitudinal data analyses, no statistically significant differences could be seen between the three fitness groups regarding symptoms of depression and/or anxiety over time, though time in itself seem to be associated with symptom reduction (Table 4).

#### Cardiorespiratory fitness and sleep

**Fig. 2:**
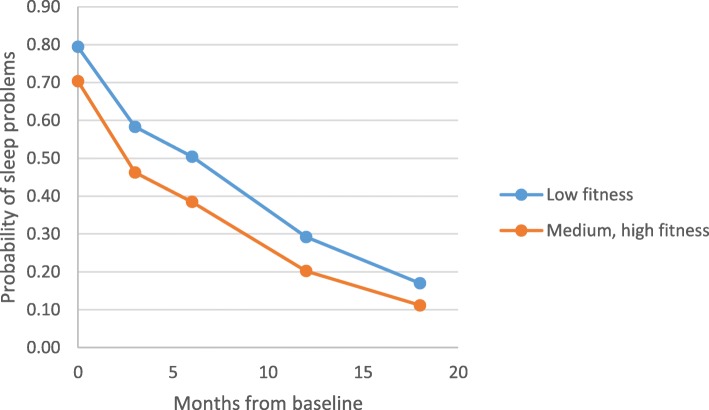
Over time associations between aerobic capacity/fitness level (binary outcome) and sleep, adjusted for identified confounders

Time stand-alone was also statistically significantly associated with fewer sleeping problems over time, i.e. sleep was improved over time regardless of cardiorespiratory fitness level. Furthermore, no significant associations could be detected regarding fitness level and sleep over time (type III *p* = 0.281). Interactions between time and fitness were not statistically significant, and none of the confounders seem to have influenced the results. However, it should be noted that analysis of the binary variable sleep problems at some time points had rather few cases (sleep disturbances = 1) in some of the fitness groups. These results should therefore be interpreted as merely rough indications of this association (Fig. 2).

#### Self-reported physical activity and symptoms of ED

**Fig. 3:**
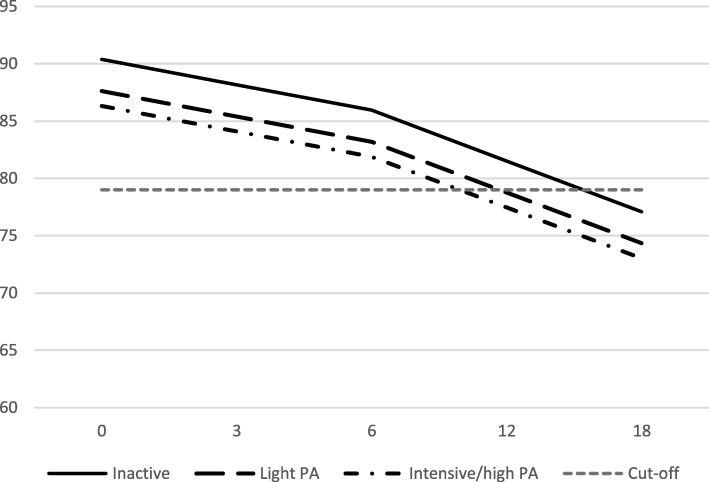
Self-reported physical activity levels and associations with symptoms of stress-related exhaustion/burnout over time. The Figure shows the Shirom-Melamed Burnout Questionnaire (SMBQ) clinical cut-off of 79. PA = physical activity

The analysis showed that self-reported physical activity levels were statistically significantly associated with symptoms of ED over time (regression coefficient − 3.4 and − 4.9, respectively, for the “light physical activity” and the “high/intensive physical activity” level compared with the “inactive/low physical activity” level; type III *p* = 0.012) although no statistically significant interactions could be seen between time and physical activity (Fig. 3).

#### Self-reported physical activity and depression and anxiety

Symptoms of depression and/or anxiety were not statistically significantly associated with self-reported physical activity levels, however; time alone was statistically significantly related to symptom reduction (data not shown).

#### Self-reported physical activity and sleep

The levels of self-reported physical activity were not statistically significantly associated with sleep disturbances over time (data not shown).

## Discussion

Our main results showed that cardiorespiratory fitness level was significantly associated with symptoms of ED over time. Moreover, symptoms of depression and anxiety and sleep disturbances were not related to differences in fitness level over time. The results also demonstrated that the most favorable development with respect to reduction in ED symptoms over time was seen among those with medium cardiorespiratory fitness, compared with those having “low” and “high” cardiorespiratory fitness.

With respect to self-rated physical activity the results showed that patients defining themselves as having a high physical activity level were also the ones who showed the best improvement over time with respect to symptoms of ED, however, patients with a high physical activity level also reported fewer symptoms at baseline, and therefore no interaction between self-reported physical activity level and time was present in this case. Baseline data on cardiorespiratory fitness level revealed no statistically significant differences between the different fitness levels apart from the fact that patients with “high fitness” were older, had a lower BMI and had a higher education level compared with those with a low fitness level. Moreover, baseline data on self-rated physical activity levels disclosed a significant association between low levels of physical activity, and longer symptom duration, poorer sleep quality, and more symptoms of exhaustion/burnout. Finally, all three fitness levels reduced their symptoms of stress-related exhaustion during the follow-ups, indicating the importance of time for recovery from symptoms related to stress-related exhaustion disorder.

Over time, numerous studies have been conducted to elicit the beneficial effects of physical activity on different health outcomes including mental disorders [14, 33–35]*.* Regarding mental disorders, most attention has until now been focused on exploring the effects of physical activity on major depression [11, 36–38]. To our knowledge the present study is the first study performed in a well-defined patient group clinically diagnosed with stress-related exhaustion using both objective and subjective physical activity exposure measures. Although other researchers have conducted studies touching this problem area most of these studies have been performed among patient groups with a broader definition of “common mental disorders”. Results from these studies point to beneficial effects of vigorous physical activity on stress reduction, mental health, and sleep, especially in participants reporting high perception of stress [39, 40]*.* Our findings revealed, however, that having medium fitness level, compared with a low or a high fitness, was associated with a more sustainable reduction in stress-related exhaustion symptoms over time. This is in line with our clinical experience and in concordance with a recently presented study whose authors conclude that lighter forms of physical activity are as effective as more intensive exercise training in reducing symptoms of mild to moderate depressive disorder (MDD) [41]. Although symptoms of ED were the major target outcome in the present study, the comparison with studies of patients diagnosed with depression is still very relevant since a high percentage (67%) of patients diagnosed with ED suffer from comorbidity with depression and/or anxiety disorders [2]. These results clearly stress the importance of recognizing the need for differentiation in recommendations regarding the dose of physical activity between individuals with light perceived stress levels and patients with clinically diagnosed stress- related exhaustion disorder. Also in line with the results of this study, our research group have previously elucidated that a physical activity level meeting the recommendations from the ACSM, individually tailored over time during rehabilitation, regarding frequency, duration and intensity, was sufficient to reduce symptoms connected to ED over a sustained period of time (18 months) [15]. The concept of an existing optimal, rather than maximal, dose of physical activity for patients with stress-related exhaustion disorder has not been sufficiently investigated in the scientific literature. Hence, the results from our study, indicating that the most sustained reduction in symptoms over time was obtained in the medium fitness group, might support the existence of an optimal dose among patients with stress-related exhaustion/burnout. Moreover, our findings are in concordance with results from another study [36] focusing on the impact of different intensities of physical activity on wellbeing among patients with mental disorders. Its authors conclude that, when it comes to improving mental health disorders, also light intensity activities should be considered as an alternative, since the axiom of “the more physical activity during a certain time the better” does not necessarily lead to better health and wellbeing among this patient group, partly because it does not seem to be applicable to all individuals in this patient group [42, 43]. One plausible explanation might be the over-representation of the personal trait “perfectionism” bordering to compulsion, previously identified in this patient group (personal communication). In combination with overcommitment in the working life as well as in the private life [44, 45] and an established sustained lack of energy due to lack of recuperation in this patient group over a long time period [3] might explain why the patients with medium fitness show a more sustained reduction of ED symptoms over time. The “commitment” with respect to physical activity “ambitions” among those ED patients with medium fitness level is more likely to correspond to their energy level and thus constitute a better prerequisite for sustainability over time.

In many of the mentioned studies, categorization of patients into different cardiorespiratory fitness groups were based solely on self-reported physical activity data, while the present study focuses on objectively measured physical activity data. Using an objective measure to estimate the actual cardiorespiratory fitness level increases the validity, primarily the predictive validity, since self-reported data on the extent of performed physical activity as an accurate and precise measure have been questioned in the scientific literature, inter alia because of all the attendant biases [46, 47]. Atienza and co-workers [48], after scrutinizing the complexity of this discussion, concluded that objective and subjective measurement of physical activity seem to capture different features of physical activity connected to different health outcomes and should consequently be used in combination to better identify and optimize individual dose recommendations for physical activity in clinical practice [48]*.*

Underlining this recommendation, Lindwall and co-workers have concluded that self-reported data may be equally good as objectively measured data in predicting the risk of future common mental health disorders [49]. However, their study was conducted in a variety of participants still at work and not reporting any major mental issues, and the possibility that patients diagnosed with stress-related ED will respond differently from “healthy” individuals with respect to both cardiorespiratory fitness and self-reported physical activity data is quite high. The increased likelihood that patients would comply with an individually tailored physical activity dose might also generate a more sustainable activity level over a long time period, since also the positive short-term effects of being regularly physically active seem to accumulate with time [50].

Regarding the non-significant results concerning longitudinal associations between sleep and cardiorespiratory fitness levels, our results are somewhat inconclusive in comparison with other studies that support the theory that fitness level is associated with improved sleep [51, 52]*.* However, these studies were performed among participants from the general population and, as mentioned before, it is likely that patients with a severe mental illness such as ED will react differently from mentally healthy individuals. Moreover, one of the studies had a cross-sectional design, which makes it impossible to draw any conclusions about causality.

As previously reported in the results, nearly all our included patients (95%) scored above the cut-off for ED at their first visit to our clinic. This suggests that the investigated patient group were clinically homogeneous, confirming that almost all the patients could be classified as severely affected by symptoms of ED, and were therefore more “resistant” to comply with the physical activity intervention and endure with it over time. The difficulties in encouraging patients with severe mental disorders to be more physically active have previously been elucidated by Daumit et al. [53]. However, when physical activity is introduced and dosed individually, substantially increased levels of exercise can be achieved in patients with severe symptoms of ED through general comprehensive instructions, in combination with or without a coach-led exercise programme, as demonstrated in one of our previous studies [54]. Regarding depression, anxiety and sleep disturbances, no statistically significant differences in symptoms over time could be detected with respect to fitness level; however, symptoms of depression and anxiety diminished more rapidly over time compared with symptoms of ED [2]. As we have pointed out in the Results, the found lack of association between cardiorespiratory fitness levels and sleep disturbances may be due to lack of power in some of the analyses, and consequently these results should be interpreted with caution. Previous studies, though performed on healthy populations, have suggested a positive impact of high fitness on sleep quality [55, 56].Concerning self-reported physical activity data, a possible explanation for the discrepancies between objectively measured vs. self-reported physical activity data and the outcome measures could be related to the established problem of bias associated with self-reports, often resulting in an overestimation of the amount of physical activity performed, as demonstrated by the results from a large study performed on a general population sample in Sweden. In that study, by Ekblom and co-workers [39], the authors conclude that there is low to moderate concurrent validity between self-reported and objectively measured physical activity data, showing an overestimation of time spent on physical activity, compared with time spent on sedentary “activities”. Therefore, a large risk for misclassifications exists when solely relying on self-reported data [46]*.*

In line with this, another study revealed that individuals categorized as belonging to low socio-economic areas had a 12% lower fitness level than individuals from higher ranked socioeconomic areas [57] indicating that a more prolonged and severe burden of disease might affect the way patients rate their physical activity level.

### Strengths of the study

One of the strengths of this study is the longitudinal design. The numerous follow-up occasions gave us the opportunity to collect robust data. This is crucial to be able to draw causal inferences from gathered data. Another strength is the use of both self-reported and objectively measured exposure data, in this case physical activity data. Potential drawbacks of using self-reported exposure data have been discussed above. A further advantage is that the patient group in focus were all diagnosed by a medical doctor with documented competence in diagnosing mental disorders including stress-related exhaustion/burnout. Hence, the risk of misclassification with respect to the diagnosis was minimal. Our study group were homogeneous regarding symptom severity and comorbidity, and the results are therefore especially robust and valid for this patient group.

### Limitations

With respect to the analysis performed, regarding sleep disturbances, some of the follow up analysis comprised rather few cases, as previously mentioned in the Results; therefore, the interpretation regarding sleep and fitness in this patient group should be considered tentative. Regarding the risk of any major carryover effects on the results pertaining from the intervention part of the study (interrupted due to several unpredictable events effecting the power of the RCT-study), might be considered as unlikely to occur due to the fact that all patients included in this here study received the basic treatment irrespective of fitness level and thus received the same information during the same relevant time frame. A consort diagram with detailed information about the original RCT-study is attached to this paper as a supplementary appendix. A final limitation was that only women participated in the study, the results should therefore be interpreted with this limitation in mind, when it comes to generalizability in a mixed population.

## Conclusions

The main conclusion from this study is that a medium cardiorespiratory fitness over time was positivity associated with a more sustained reduction in symptoms of ED in women diagnosed with ED compared to those having low or high cardiorespiratory fitness. The clinical implication following the interpretation of these results is that an individual recommendation based on a medium level of physical activity in line with the recommendations from ACSM (American College of Sports Medicine) is preferable compared to recommendations including more vigorous physical activity in order to restore and sustainably reduce symptoms of ED.

## Data Availability

The datasets used and/or analysed during the current study are available from the corresponding author on reasonable request.

## Abbreviation

ED: Exhaustion disorder
